# Rational Design of a Potent Two-Phage Cocktail Against a Contemporary *Acinetobacter baumannii* Strain Recovered from a Burned Patient at the Lausanne University Hospital

**DOI:** 10.3390/v17111441

**Published:** 2025-10-29

**Authors:** Hugues de Villiers de la Noue, Gwenaëlle Golliard, Xavier Vuattoux, Grégory Resch

**Affiliations:** Laboratory of Bacteriophages and Phage Therapy, Center for Research and Innovation in Clinical Pharmaceutical Sciences (CRISP), Lausanne University Hospital (CHUV) and University of Lausanne (UNIL), 1005 Lausanne, Switzerlandxavier.vuattoux@unil.ch (X.V.)

**Keywords:** *Acinetobacter baumannii*, bacteriophage, phage therapy, *Galleria melonnella*, colistin, phage–antibiotic synergy, phage resistance, phage cocktail

## Abstract

*Acinetobacter baumannii* is a critical public health threat, particularly with the rise in multidrug-resistant (MDR) and extensively drug-resistant (XDR) strains that limit treatment options. Phage therapy, which uses bacteriophages to target bacteria, offers a promising alternative. We isolated an XDR strain (Ab125) from a burn wound infection and screened 34 phages, identifying vB_AbaM_3098 as the only effective candidate. However, resistance rapidly emerged, producing a derivative strain (Ab139). Interestingly, Ab139, though resistant to vB_AbaM_3098, became susceptible to six previously inactive phages. While various potential determinants were identified through comparative genomics and proteomics, the mechanism causing phage resistance to vB_AbaM_3098 and simultaneous susceptibility to other phages remains to be elucidated. Among the six new candidates, vB_AbaM_3014 was the most promising. While each phage alone allowed bacterial regrowth, combining vB_AbaM_3098 and vB_AbaM_3014 completely suppressed Ab125 growth. In a *Galleria mellonella* infection model, this cocktail achieved 90% survival after five days compared to 0% in untreated controls. Notably, the cocktail combined one phage with modest activity and another inactive phage against the parental strain; together, they produced strong bactericidal effects. These findings highlight both the complexity of phage cocktail design and their promise as adjunct therapies against drug-resistant *A. baumannii*.

## 1. Introduction

*Acinetobacter baumannii* is an aerobic, Gram-negative, and non-fermentative opportunistic pathogen often implicated in hospital-acquired infections, particularly among immunocompromised individuals and patients receiving mechanical ventilation in intensive care units (ICUs) [1,2]. In 2018, the World Health Organization (WHO) designated carbapenem-resistant *A. baumannii* (CRAB) as one of the highest priorities for new antibiotic research and development. This emphasizes its significance as one of the most prevalent multidrug-resistant (MDR) pathogens in existence, alongside other pathogens of the “ESKAPE” group (*Enterococcus faecium*, *Staphylococcus aureus*, *Klebsiella pneumoniae*, *Pseudomonas aeruginosa*, and *Enterobacter* spp.) [3]. Similarly, the 2022 Global Antimicrobial Resistance and Use Surveillance System (GLASS) report highlights worrying levels of carbapenem and aminoglycoside resistance in Acinetobacter species, with a significant increase in extensively drug-resistant (XDR) isolates [4]. Resistance has increased to such an extent that 50% of *A. baumannii* clinical isolates worldwide are now MDR [5]. This resistance, which developed at an alarming rate, renders many therapeutic options ineffective and limits alternatives for treating infected patients. The threat posed by *A. baumannii* is exacerbated by its ability to survive in hostile hospital environments, where it can easily be transmitted between patients. In this context, the management of *A. baumannii* infections becomes complex, requiring not only appropriate antimicrobial treatments but also strict control measures to limit its spread. One promising complementary treatment approach is phage therapy, which relies on the capacity of bacterial viruses named bacteriophages (phages) to infect and kill bacterial cells. Phages are the most widespread biological entities in our biosphere, playing an essential role in the evolution, diversity, and regulation of bacterial populations. Oceans, for example, are home to at least 10 million times more bacterial viruses than there are stars in the universe [6]. They are found wherever bacteria thrive, including in sewage, rivers, and the digestive tracts of animals [7]. Most phages are composed of an icosahedral head, a sphere with 20 flat faces made of proteins, and contain a nucleic acid genome, to which a protein tail is attached [8]. Phages adsorb onto the surface of the target bacterium with their tail fibers and spikes. Subsequently, the phage genome is injected into the cytoplasm of the bacterial cell; in the case of lytic phages, it directly hijacks the bacterial DNA and protein synthesis machinery in order to replicate. Following a variable latent period, the newly formed phages burst out of their bacterial hosts, causing bacterial lysis. The phage progeny, which can number in the hundreds per bacterium, then proceed to infect new host bacteria [9]. As phages have evolved to infect specific bacteria, they are harmless to mammalian cells and allow for targeted treatment, minimizing microbiome disruption [10], making them ideal candidates for therapy. Shortly after their discovery by D’Hérelle in 1917, phages were used in therapy until the democratization of antibiotics and the decline of interest in their use in the West in the 1970s [11]. Since the early 2000s, with the intensification of antibiotic resistance, phage therapy has been reintroduced into clinical treatment, with many cases of compassionate treatment [12] and clinical trials underway [13,14]. It is worth noting that a high-profile compassionate case for treating a disseminated *A. baumannii* infection paved the way for phage therapy as an adjuvant treatment to antibiotics in XDRAB infections [15]. In this study, we thoroughly developed and characterized the efficacy of a two-phage cocktail against an XDR *A. baumannii* isolate (designated Ab125), obtained from a patient with a burn wound infection at CHUV.

## 2. Materials and Methods

### 2.1. Bacterial Strains, Bacteriophages, and Antibiotics

This study focuses on an XDRAB strain, Ab125, isolated from the infected burn wounds of a patient at Lausanne University Hospital (CHUV) and its phage 3098-resistant derivative (Ab139). *A. baumannii* strain Ab7 was used to amplify and titrate phage 3014. All strains were grown aerobically for 16–24 h in Luria broth (LB, Sigma-Aldrich, Saint-Louis, MO, USA) at 37 °C and 200 rpm. Glycerol stocks were prepared from 750 μL of overnight cultures supplemented with 20% glycerol (Sigma-Aldrich) and stored at −80 °C. Glycerol stocks were inoculated directly onto Luria agar (LA) plates before being incubated overnight at 37 °C aerobically. After incubation, the plates were stored at 4 °C until further use for a maximum period of two weeks. A collection of 34 lytic *A. baumannii* phages previously gathered in the laboratory was investigated. Solutions of ceftazidime (CTZ) and colistin (COL) (Toronto Research Chemicals, North York, ON, Canada), ciprofloxacin (CIP) and monocycline (MIN) (Sigma-Aldrich), gentamicin (GEN) (Ratiopharm, Ulm, Germany), and imipenem (IPM) (Labatec, Meyrin, Switzerland) were prepared and stored according to the manufacturer’s recommendations.

### 2.2. Minimum Inhibitory Concentration (MIC)

The MIC of antibiotics was determined for Ab125 using the broth macrodilution method [16]. For this purpose, Ab125 was grown aerobically overnight at 37 °C and 200 rpm and diluted to a 600 nm optical density (OD_600nm_) of 0.1 in Mueller–Hilton broth (MH) the next morning. We filled 5 mL test tubes with 1 mL of MH supplemented with serial dilutions of each antibiotic and 1 mL of the diluted Ab125 culture. Tubes were incubated aerobically without shaking for 17–20 h at 37 °C. After incubation, 200 μL of a 0.1% solution of 2,3,5-triphenyltetrazolium chloride (TTC) (Sigma-Aldrich) was added to each tube. The tubes were then incubated for 2–3 h at 37 °C to allow bacterial conversion of TTC to red formazan via dehydrogenases in viable bacterial cells. The MIC was defined as the lowest concentration of antibiotic required to inhibit red formazan production. The results obtained were used to determine the concentrations of each antibiotic in the checkerboard assay.

### 2.3. Phage Amplification and Titration of Phage Suspensions

Phage amplification was performed in 20 mL of fresh LB inoculated with 200 μL of an Ab7 (for phage vB_AbaM_3014) or an Ab139 (for phage vB_AbaM_3098) overnight culture incubated at 37 °C and 200 rpm. At an OD_600nm_ of 0.25–0.35, 200 μL of a single-phage suspension (1 × 10^6^ PFU/mL) was added to the bacterial suspension. Following incubation at 37 °C with agitation at 200 rpm, the phage lysate was subjected to centrifugation at 8000 rpm for 30 min to remove surviving bacteria and large bacterial debris. The supernatant containing the amplified phages was filtered using a 10 mL Omnifix syringe (B. Braun, Saint-Cloud, France) and a 0.45 μm filter (Sarstedt, Nümbrecht, Germany). The phage filtrate was stored at +4 °C until further use. The phage titer in the filtrate was determined using a classical double-layer assay (DLA). Briefly, the phage suspension was serially diluted in NaCl 0.9%. In total, 200 μL of an overnight culture of Ab7; 100 μL of a diluted phage suspension; and 5 mL of soft LA were mixed and poured on top of an LA plate and incubated at 37 °C overnight after solidification of the soft-agar layer. PFUs were enumerated on each plate, and phage titer was determined accordingly. Each experiment was performed in triplicate. Phage stability at 4 °C was assessed every two weeks by performing a phage titer measurement through the diluted drop test assay (DDT). Briefly, 20 mL soft LA was inoculated with 350 µL of an overnight culture of the corresponding *A. baumannii* strain. The mixture was poured into an empty Petri dish. Once the agar solidified, 5 μL of the serially diluted phage suspensions was deposited on top of the agar layer. After overnight incubation at 37 °C, single PFUs were enumerated at low concentrations with the phage titer calculated accordingly. Each experiment was performed in triplicate.

### 2.4. Genome Extraction, Sequencing, and Annotation

Full genome sequencing was performed by the Laboratory of Molecular Diagnostics, Metagenomics and Biosafety, Institute of Microbiology, University of Lausanne. Phage 3014 and bacterial DNA were extracted using the MagNA Pure 96^®^ (MP96) nucleic acid extraction system (Roche, Basel, Switzerland). Phage 3098 DNA was extracted using a different method, as commercial kits did not work. Briefly, 750 µL of phage 3098 at 1 × 10^10^ PFU/mL was incubated with 0.1 mg RNase (Promega, Madison, WI, USA) and DNase (Promega) at 37 °C for 30 min, before incubating at 65 °C for 10 min and 95 °C for 10 min. In total, 250 µL of lysis buffer (133 µL EDTA 0.5 M; 8.3 µL Tris 1 M; 8.3 µL ddH_2_O; 75 µL SDS 10%; 25 µL proteinase K (Promega)) was then added, and the sample was incubated at 55 °C for 90 min. Following capsid digestion, DNA was extracted using phenol–chloroform–isoamylalcohol (PCA, Merck, Buchs, Switzerland). Library preparation was performed with the Nextera XT DNA^®^ Sample Preparation kit (Illumina, San Diego, CA, USA), and sequencing was conducted with 150 bp paired-end reads on an Illumina MiSeq^®^ sequencer (Illumina). Bacterial read quality was assessed with FastQC version 0.11.08 [17]. Trimmomatic version 0.32 [18] was used to filter low-quality reads and reads shorter than 150 bp. Reads were assembled with SPAdes genome assembler v.3.15.4 [19], using k-mer sizes from 43 to 127 bases. To select the best assembly, QUAST v.5.3.0 [20] was used, prioritizing the assembly with the fewest contigs and the largest N50. Contigs shorter than 1000 bp and those with k-mer coverage below 2× were excluded. Gene annotation was conducted with Prokka v.1.13 [21]. Variant calling was performed using GATK HaplotypeCaller v.4.1.4 [22] based on read mapping to the Ab125 genome as a reference with BWA v.0.7.17 [23].

Phage read quality was evaluated using FastQC v.0.11.08 [17]. Fastp version 0.23.2 [24] was applied to filter low-quality reads, and Unicycler v.0.4.8 (with --depth_filter 0.1) [25] was used for assembly. Phage genome annotation was performed with Pharokka v.1.3.2 [26]. Taxonomic classification of the assembly was conducted with the contig annotation tool (CAT) v.5.3 [27], using the NCBI viral sequence database (accessed on 7 February 2023). Phage lifestyle prediction was performed with Bacphlip v.0.9.3 [28] and PhageAI (https://www.phage.ai/). ResFinder (http://genepi.food.dtu.dk/resfinder, accessed on 15 July 2025) and the blast search tool of the virulence factor database (VFDB, https://www.mgc.ac.cn/VFs/, accessed on 15 July 2025) were used to identify any genes coding for antibiotic resistance and virulence determinants, respectively.

### 2.5. Electron Microscopy

Morphology of phage particles was studied using a JEOL 100C electron microscope (Jeol, Akishima-Shi, Tokyo, Japan). Phage suspension (1 × 10^9^–1 × 10^10^ PFU/mL) was transferred onto carbon-coated copper grids for 30 s to allow particles to settle before being stained with 1% uranyl-acetate for 40 s. Filter paper was used to wipe away any excess sample. Grids were examined at different magnifications.

### 2.6. Turbidity Assay and Virulence Index Determination

While an overnight culture of *A. baumannii* was diluted in fresh LB at 1 × 10^6^ CFU/mL, phages were serially diluted in fresh LB from 1 × 10^8^ PFU/mL to 1 × 10^2^ PFU/mL. Then, 10 μL of bacterial suspension was added to wells of a 96-well plate with 10 μL (phage alone) or 2 × 5 μL (two phages in combination) of phage suspensions at different titers to test multiplicity of infection (MOI) from 100 to 1.00 × 10^−4^. In total, 180 μL of LB was added to each well; 200 μL of LB was used as a control for growth medium contamination, and 190 μL of LB was added to 10 μL of bacterial suspension for the bacterial growth control. Each experiment was performed in triplicate. We placed 96-well plates in a microplate reader (FluoStar Galaxy, BMG LABTECH, Champigny-sur-Marne, France) at 37 °C, and absorbance at OD_600nm_ was measured every 10 min for 48 h with shaking for 3 s before each measurement. Growth curves were plotted and analyzed using GraphPad Prism v.10.1.2 (GraphPad, Boston, MA, USA). The method described by Storms et al. [29] was used to assess phage virulence, which is based on the following three parameters: the virulence index (*V_p_*), which quantifies phage virulence against a given bacterial strain; local virulence (*vi*) that evaluates the killing potential at a given MOI; and the *MV*_50_, which represents the MOI at which the phage reaches 50% of the maximum virulence a phage can theoretically reach, which is 1. These parameters were determined for phages alone and phage cocktails on *A. baumannii* clinical strains over an MOI range from 1 × 10^2^ to 1 D7; 10^−4^. Briefly, for each MOI, turbidity assays at OD_600nm_ were performed to acquire turbidity data covering the period between t0 (i.e., phage challenge) and the start of the stationary growth phase observed when no phages were added. Growth curves were drawn from these data for each MOI using GraphPad Prism v.10.1.2, and the area under the curve (AUC) was calculated for each condition. *vi* was then calculated using the following formula: *v_i_* = 1 − *A_i_*/*A*_0_. Here, *A_i_* is the AUC of the phage-infected bacterial culture at a given MOI, and *A*_0_ is the AUC of the control condition, i.e., the bacterial strain without phages (maximum AUC). Using the determined *v_i_*, the local virulence was plotted in relation to MOI. The virulence index (*V_P_*) for each phage was extracted from these curves with the following formula: *V_P_* = *A_P_*/*A_max_*. Here, *A_P_* corresponds to the area under the virulence curve, and *A_max_* relates to the theoretical maximum area under the virulence curve. When possible, *MV*_50_ was deduced from the virulence curve as the MOI for *vi* = 0.5.

### 2.7. Synogram

The modified checkerboard assay in the 96-well plate previously described and synography assays were used to investigate the effect of different antibiotics in combination with the 3014/3098 phage cocktail against Ab125 [30]. The tested antibiotic concentrations were determined for each antibiotic based on the predetermined MIC. The tested phage cocktail MOIs were determined according to the minimal inhibitory MOI (MIM) observed in the turbidity assays. Experiments were performed in MH using a starting bacterial inoculum of ca. 1 × 10^5^ CFU/mL. The 96-well plates were incubated aerobically for 17–20 h at 37 °C, and 20 μL of a 0.1% TTC solution was added to each well. OD_483nm_ was measured for each well, and we calculated the Ab125 growth percentages compared to the growth control using the following formula:

$$\%\;Ab125\;growth\;=\frac{Bacterial\;Growth\;Mean\;-\;Sterility\;Control\;Mean}{Bacterial\;Growth\;Control\;Mean}\;\times 100$$

The concentrations of each treatment (MIC and MIM) in the combination that most effectively inhibited the formation of red formazan were determined and used to calculate the fractional inhibitory concentration index (FICI) according to the following formula:

$$FICI={FIC}_{antibiotic}+{FIC}_{cocktail}=\frac{{MIC}_{antibiotic+cocktail}}{{MIC}_{antibiotic}}+\frac{{MIM}_{cocktail+antibiotic}}{{MIM}_{cocktail}}$$

A FICI < 0.50 indicated a phage/antibiotic synergy, 0.50 ≤ FIC < 1.0 indicated an additive effect, 1.0 ≤ FICI < 2.0 indicated indifference, and FICI ≥ 2.00 indicated antagonism [31].

### 2.8. Time–Kill Assay

Ab125 grew aerobically overnight in MH at 37 °C and 200 rpm. The culture was then diluted to 1 × 10^5^ CFU/mL in fresh MH. In total, 2 × 10^5^ CFU of Ab125 was challenged with either 3 µg/mL colistin, 50 µg/mL colistin, the phage cocktail at MOI = 1 × 10^−3^, colistin 3 µg/mL + the phage cocktail at MOI = 1 × 10^−3^, or colistin 50 µg/mL + the phage cocktail at MOI = 1 × 10^−3^. Growth control without antimicrobials was performed. Mixtures were then incubated at 37 °C for 24 h, and 100 µL aliquots were taken at 1 h, 3 h, 5 h, 8 h, and 24 h post-challenge. To avoid carry-over, samples were immediately diluted and plated on LA, and CFUs were counted after 24 h incubation at 37 °C. Graphs were generated with GraphPad Prism v.10.1.2.

### 2.9. Proteomic Analyses

Ab125 and Ab139 were grown under the same conditions in LB at 37 °C with 200 rpm shaking until an OD_600nm_ of 0.7–0.8 was obtained. The cells were collected by centrifugation at 10,000× *g* for 10 min and digested following the SP3 method [32] with 50 mg/mL magnetic Sera-Mag Speedbeads (Cytiva^®^, Malborough, MA, USA). Briefly, samples were diluted with SP3 buffer (2% SDS, 10 mM DTT, 50 mM Tris, pH 7.5) and heated for 10 min at 75 °C. Proteins were then alkylated with 32 mM (final concentration) iodoacetamide for 45 min at room temperature (RT) in the dark. Beads were added at a ratio of 10:1 (*w*:*w*) to samples, and proteins were precipitated on beads with ethanol (60% final concentration). After three washes with 80% ethanol, beads were digested in 50 µL of 100 mM ammonium bicarbonate with 0.4 µg of trypsin (Promega). After 1.5 h of incubation at 37 °C, the same amount of trypsin was added to the samples for an additional 1.5 h of incubation. The supernatant was then recovered, transferred to new tubes, acidified with formic acid (0.5% final concentration), and dried by centrifugal evaporation. Two sample volumes of isopropanol containing 1% trifluoroacetic acid (TFA) were added to the digests, and the samples were desalted on a strong cation exchange plate (Oasis MCX; Waters Corp., Milford, MA, USA) by centrifugation. After washing with isopropanol/1%TFA and 2% acetonitrile/0.1% formic acid (FA), peptides were eluted in 150 µL of 40% acetonitrile (MeCN), 59% water, 1% (*v*/*v*) ammonia, and dried by centrifugal evaporation.

Tryptic peptide mixtures were injected into a Vanquish Neo nanoHPLC system interfaced with a nanospray Flex source to a high-resolution Orbitrap Exploris 480 mass spectrometer (Thermo Fisher Scientific, Bremen, Germany). Peptides were loaded onto a trapping microcolumn PepMap100 C18 (5 mm × 1.0 mm ID, 5 µm, Thermo Fisher) before separation on a C18 custom packed column (75 µm ID × 45 cm, 1.8 μm particles, Reprosil Pur, Dr. Maisch), using a gradient that ranged from 2% to 80% acetonitrile in 0.1% formic acid for peptide separation at a flow rate of 250 nL/min (total time: 130 min). Full MS survey scans were performed at a 120,000 resolution. A data-dependent acquisition method controlled by Xcalibur v.4.3 software (Thermo Fisher Scientific) was used to optimize the number of precursors selected (“top speed”) with charge 2+ to 5+ while maintaining a fixed scan cycle of 2 s. Peptides were fragmented by a higher-energy collision dissociation (HCD) with a normalized energy of 30% at 15,000 resolution. The window for precursor isolation was 1.6 *m*/*z* units around the precursor, and selected fragments were excluded for 60 s from further analysis. Data files were analyzed with MaxQuant 2.5.1.0 [33] incorporating the Andromeda search engine [34]. Cysteine carbamidomethylation was selected as a fixed modification while methionine oxidation and protein N-terminal acetylation were specified as variable modifications. The sequence database searched was the *A. baumannii* strain ATCC17978 total proteome based on the UniProt database (www.uniprot.org, accessed on 24 July 2024), and a “contaminant” database containing the most common environmental contaminants and enzymes used for digestion (keratins, trypsin, etc.). Mass tolerance was 4.5 ppm for precursors (after recalibration) and 20 ppm for MS/MS fragments. Both peptide and protein identifications were filtered at 1% FDR relative to results against a decoy database built by reversing protein sequences. All subsequent analyses were performed with an in-house developed software tool (available on https://github.com/UNIL-PAF/taram-backend, accessed on 15 July 2025). Contaminant proteins were removed, and quantity values for protein groups generated by MaxQuant were log2-transformed. After assignment to groups, only proteins quantified in at least 3 samples of one group were kept. Missing values were imputed based on a normal distribution with a width of 0.3 standard deviations (SDs), downshifted by 1.8 SD relative to the median value. Student’s *t*-tests were carried out for various conditions, with Benjamini–Hochberg correction for multiple testing (*Q*-value threshold < 0.05). Imputed values were later removed. All raw MS data together with raw output tables are available via the Proteomexchange data repository (www.proteomexchange.org, accessed on 1 April 2025) with the accession No. PXD062593.

### 2.10. Bacterial Virulence Testing in Galleria mellonella

Ab125 and Ab139 grew overnight in LB at 37 °C and 200 rpm. The strains were centrifuged for 10 min at 4 °C and 6000× *g* and resuspended in saline. The bacterial suspensions were diluted in saline at 1 × 10^5^, 1 × 10^6^, and 1 × 10^7^ CFU/mL. Bacterial concentration in the suspension was verified by performing serial dilution and CFU counting in triplicate. Late-stage *G. mellonella* wax moth (Bernhard-Fishing, Wichtrach, Switzerland) was stored at 15 °C for a maximum of one week. Before each experiment, larvae weighing 250–350 mg were placed at 37 °C overnight in the dark. Surviving larvae were injected again on the same day. Three groups of larvae were injected in the last right pseudopod with 10 μL of Ab125 or Ab139 suspension at different concentrations (1 × 10^5^, 1 × 10^6^, and 1 × 10^7^ CFU/mL, n = 10–15). For control purposes, when testing the impact of the injection procedure on larval survival, one group of larvae was not injected, and another was injected in the last right pseudopods with 10 μL of sterile saline. To perform these injections, 0.5 mL insulin syringes equipped with a 30 G × 8 mm needle (BD Biosciences, Franklin Lakes, NJ, USA) were used. Larvae were incubated in Petri dishes at 37 °C in the dark with the bottom lined with filter paper. Larvae survival was monitored every 12 h to 24 h for four days. Larvae were considered dead when they did not respond to physical stimulus. Survival curves were plotted and analyzed using GraphPad Prism v.10.1.2 software. Curves were compared with the log-rank (Mantel–Cox) and Gehan–Breslow–Wilcoxon tests.

### 2.11. Antibacterial Activity of Phages in the Galleria mellonella Model of Infectious Diseases

Ab125 grew aerobically overnight in LB at 37 °C and 200 rpm. The next morning, the culture was centrifuged for 10 min at 4 °C and 6000× *g* and resuspended in saline at 1 × 10^6^ CFU/mL. Bacterial concentration in the suspension was verified by plating serial dilutions on LA and CFU counting in triplicate. Phages 3014 and 3098 were both diluted at 1 × 10^8^ PFU/mL in saline, and the two-phage cocktail was prepared by mixing equal volumes of both phages (with a final phage concentration of 1 × 10^8^ PFU/mL). Four groups of larvae (n = 10–15) were injected with 10 μL (i.e., 1 × 10^4^ CFU) of Ab125 in the last right pseudopod. After 30 min, the four groups were injected in the last left pseudopod with 10 μL of either saline (infection control), phage 3014 alone (1 × 10^6^ PFU, MOI = 100), phage 3098 alone (1 × 10^6^ PFU, MOI = 100), or the phage cocktail 3014 + 3098 (1 × 10^6^ PFU, MOI = 100). Several additional control groups were included to monitor the effect of the injection procedure and potential toxicity of the phages; (i) no injection; (ii) 10 μL of saline in the last right pseudopod followed by 10 μL of saline in the last left pseudopod; and (iii) 10 μL of saline in the last right pseudopod followed by 10 μL (i.e., 1 × 10^6^ PFU) in the last left pseudopod of either phage 3014, phage 3098 or phages 3014 + 3098, respectively. After injection, the larvae were placed in Petri dishes at 37 °C in the dark with the bottom lined with filter paper. Larvae survival was monitored every 12 h to 24 h for four days. Larvae were considered dead when they did not respond to physical stimulus. Survival curves were plotted and analyzed using GraphPad Prism v.10.1.2. Curves were compared with the log-rank (Mantel–Cox) and Gehan–Breslow–Wilcoxon tests.

## 3. Results

### 3.1. Ab125 Challenge with Phage 3098 Restored Susceptibility to Different Phages

**Figure 1:**
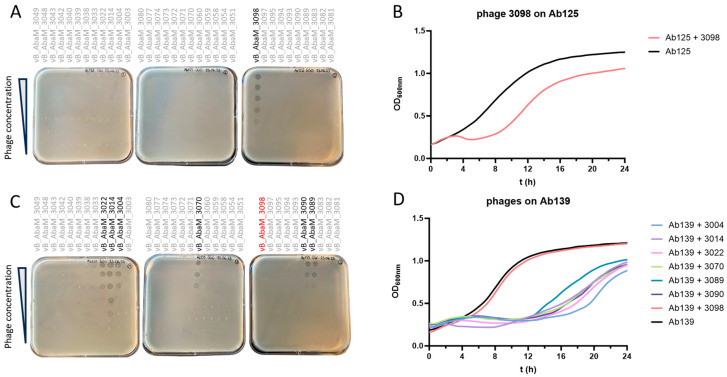
Phage susceptibility testing (PST) of Ab125 and Ab139. (**A**) Diluted drop test (DDT) for 34 phages of the CHUV collection on Ab125. The phage vB_AbaM_3098 (3098), able to lyse Ab125, is highlighted in black. (**B**) The turbidity assay on Ab125 with phage 3098 at MOI = 1. Growth of the Ab125 phage 3098-resistant clone (Ab139) starts at around 5 h (red curve). (**C**) DDT of the 34 phages against Ab139. The six phages able to lyse Ab139 are highlighted in black; phage 3098, which was unable to lyse Ab139, is highlighted in red. (**D**) The turbidity assay on Ab139 with seven selected phages alone at MOI = 1.

Amongst the 34 *A. baumannii* lytic phages previously isolated in the laboratory, only vB_AbaM_3098 (3098) proved efficient in lysing and forming PFUs on the XDRAB strain Ab125 collected from a patient’s infected burn wound (Figure 1A). However, rapid bacterial regrowth (i.e., after 5 h) was observed in the turbidity assay at an MOI of 1 (Figure 1B). Clones that survived the phage 3098 challenge in the turbidity assay were collected at 24 h and plated on Columbia agar with 5% sheep’s blood (COS). Since all colonies harbored a similar morphology, a single colony was picked for further investigation, designated as Ab139. Ab139 was found to fully resist phage 3098 in DDT (Figure 1C) and the turbidity assay (Figure 1D). However, DDT revealed that phages vB_AbaM_3004 (3004), vB_AbaM_3014 (3014), vB_AbaM_3022 (3022), vB_AbaM_3070 (3070), vB_AbaM_3089 (3089) and vB_AbaM_3090 (3090), previously inactive on Ab125, were now able to form PFUs on Ab139 (Figure 1C). Susceptibility of Ab139 to the six phages was confirmed in turbidity assays at MOI = 1, with phage 3014 performing slightly better at early time points (Figure 1D). Interestingly, while bacterial regrowth was still observed for Ab139 when challenged by each of the six phages alone, this phenomenon was delayed to 12 h (Figure 1D).

### 3.2. Comparative Genomics Between Ab125 and Ab139 Identified Genomic Variants

To investigate the mechanisms of Ab139 resistance to phage 3098 and susceptibility to the six other phages, comparative genomics was performed between the genomes of six Ab139 strains and one Ab125 strain. The full genome sequencing of Ab125 and Ab139 demonstrated extensive mean coverage, i.e., >370X and >410X, respectively. In total, 98% of the reads of Ab125 and Ab139 were attributed to *A. baumannii*, suggesting very low external contamination. In addition, >99% of Ab139 reads aligned with the assembled Ab125 genome, indicating extensive similarity between the two isolates, which was consistent since Ab139 derives from Ab125. Variant calling identified nine variants in total, amongst which three were shared by the six Ab139 clones, i.e., a deletion in an intergenic region separating two coding sequences (CDSs), which encode for hypothetical proteins (locus tags: ACSYTK_15370 and ACSYTK_15375, GeneBank accession: MGL3992130.1 and MGL3992131.1, respectively); a T > G single-nucleotide polymorphism (SNP) in a gene coding for a putative repressor protein from the LexA family transcriptional regulator (locus tag: ACSYTK_02970; GeneBank accession: MGL3989721.1); and 3795_3804delTACTAGCAATinsGACCAATAAC in a gene coding for a cell surface autotransporter adhesin Ata (locus tag: ACSYTK_07445, GeneBank accession: MGL3990584.1) (Table S1).

### 3.3. Proteome Profiling Does Not Correlate with the Identified Genomic Variants

Full proteomic sequencing identified six significantly underexpressed and one significantly overexpressed protein in Ab139 compared to Ab125 (Table S2). However, none of these differentially expressed proteins corresponded to hypothetical proteins encoded by the genes surrounding the identified intergenic SNP or the LexA or Ata protein directly impacted by a SNP or a deletion/insertion, respectively (see Section 3.2).

### 3.4. Ab139 Showed No Impaired Virulence In Vivo

The impact of acquiring resistance to phage 3098 on the Ab139 virulence was assessed in vivo in *G. mellonella.* Wax moths were injected with 10 µL of either Ab125 or Ab139 at 1 × 10^6^ or 1 × 10^7^ CFU/mL. For both Ab125 and Ab139, the inoculum killed the larvae at an insignificantly different pace to achieve 100% death at 108 h and 96 h, respectively (Figure S1).

### 3.5. A Phage Cocktail Composed of Phage 3098 and a “Non-Active” Phage Fully Inhibited the Growth of the Parental Strain Ab125

**Figure 2:**
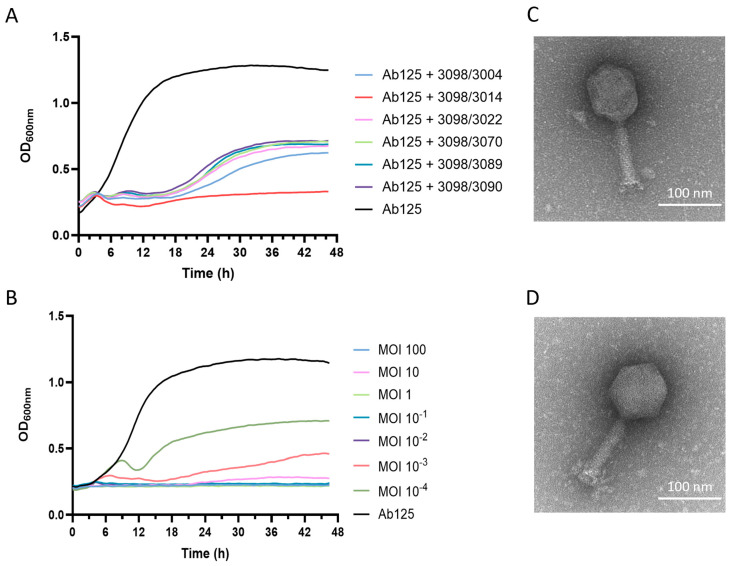
Phage cocktail assembly and characterization. (**A**) Turbidity assay on Ab125 with different phage cocktails at MOI = 1. (**B**) Turbidity assay on Ab125 with phage cocktail 3014/3098 at different MOI. (**C**) TEM image of phage vB_AbaM_3014. (**D**) TEM image of phage vB_AbaM_3098.

Phage 3098 was then combined in cocktails with one of the six phages that formed PFUs on the phage 3098-resistant strain Ab139. Turbidity assays showed that the 3098/3014 phage cocktail was the most potent combination against Ab125. Indeed, it prevented bacterial regrowth at 18 h, which was otherwise observed with all other combinations (Figure 2A). This bacterial growth inhibition was observed for up to 48 h at an MOI as low as 1 × 10^−2^ (Figure 2B).

Additionally, three parameters, i.e., the virulence index (*V_P_*), local virulence (*v_i_*), and the MOI at which the phage reached 50% of the theoretical maximum virulence, which showed full inhibition of growth without regrowth of resistant clones (*MV*_50_), were calculated for phage 3014 and 3098 alone and their combination on Ab125. Detailed virulence curves from which *V_P_* and *MV*_50_ were extracted are available in the Supplementary Materials (Figure S2). Phage cocktail 3014/3098 showed higher virulence (*V_P_* = 0.62) than phage 3014 and phage 3098 alone (*V_P_* = 0 and 0.47, respectively, Table 1). While neither phage 3014 nor 3098 reached *MV*_50_ when tested alone, this was attained with the two-phage cocktail at a low MOI of 1.30 × 10^−3^ (Table 1).

### 3.6. Both Phages 3014 and 3098 Were Suited for Phage Therapy

Regarding the high potency of the 3014/3098 combination against Ab125, phages 3014 and 3098 were further characterized by TEM and full genome sequencing. Phages 3014 and 3098 were similar in size and structure, with an icosahedral head and a medium-sized tail (Figure 2C and Figure 2D, respectively). Phage 3014 was classified as *Lazarusvirus* with a genome of 165,827 bp, harboring a G + C content of 36.73%, 268 coding sequences (CDS), and six genes encoding for tRNAs (Genbank accession No. PV081574). Phage 3098 has a genome of 55,604 bp, harboring a G + C content of 36.04%, 102 CDSs (all encoding for hypothetical proteins), and two genes encoding for tRNAs (Genbank accession No. PV081573). The closest neighbor for phage 3098 was the *Obolenskivrus* phage vB_AbaM-SPB (Genbank accession No. PP977194), with a genome identity of 91.24% over only 3% of the genome length. Both phages 3014 and 3098 were well-suited for phage therapy. Indeed, they are strictly virulent, and their genomes do not carry any gene coding for known virulence or antibiotic resistance factors.

### 3.7. The High Potency of the 3014/3098 Combination Was Confirmed In Vivo

**Figure 3:**
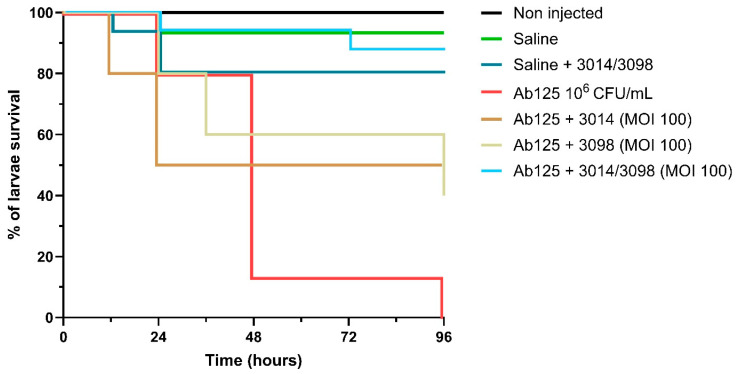
Efficacy of phage cocktail 3014/3098 on Ab125-infected *Galleria mellonella*. A volume of 5 µL of Ab125 at 1 × 10^6^ CFU/mL was injected to the larvae. Phages were administered at an MOI of 100. The percentage of survival is defined as the number of living larvae at a given time after injection divided by the total number of larvae prior to injection (N = 15). Curves were compared with the log-rank (Mantel–Cox) and Gehan–Breslow–Wilcoxon tests.

We further tested the 3014/3098 combination in the in vivo *G. mellonella* model of infectious diseases. Administration of saline alone (to mimic the injection of the bacterial inoculum) and the phage cocktail 3014/3098 only (1 × 10^6^ PFU) demonstrated no significant toxicity (92.5% and 80% survival compared to 100% with the non-injected group, respectively, *p* = 0.1574, Figure 3). While treatment with single phages at MOI = 100 already showed a significant increase in larvae survival at 96 h (50% and 43.75% survival compared to 0% in the infected control, for 3014 and 3098, respectively, *p* < 0.001), treatment with the 3014/3098 combination drastically increased survival to 86.67% (*p* < 0.001 compared to the infected control) (Figure 3).

### 3.8. The 3014/3098 Phage Cocktail Showed Additivity with Colistin In Vitro

**Figure 4:**
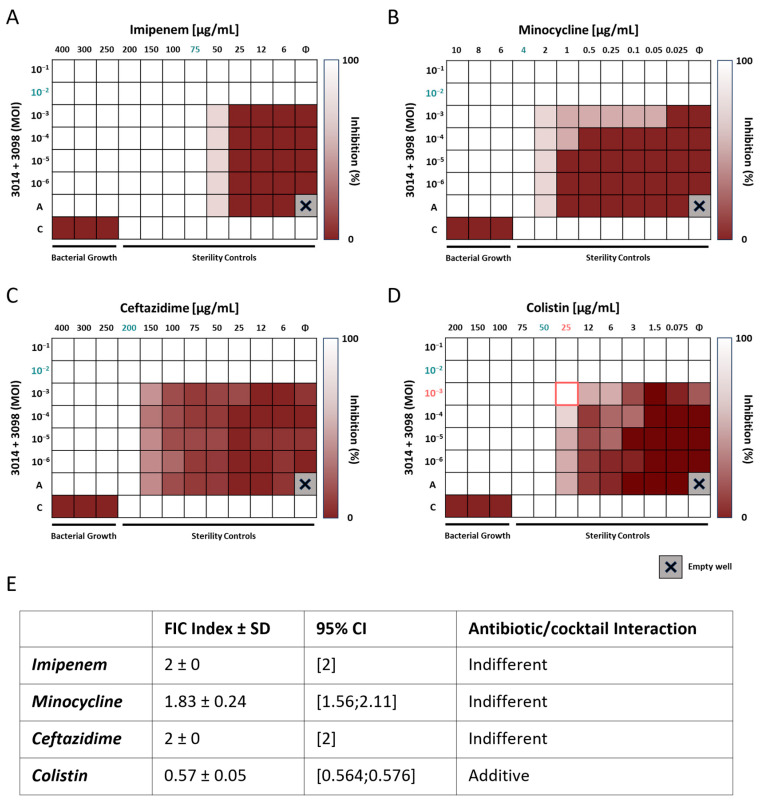
Synography assays between phage cocktail 3014/3098 and SOC antibiotics on Ab125. (**A**–**D**) Synograms illustrating the percentage of Ab125 growth inhibition (the mean of triplicates). Percentage of Ab125 growth inhibition is indicated by a gradient from dark red (no inhibition) to white (full growth inhibition). A indicates rows with antibiotics only. Φ indicates columns with the phage cocktail only. Numbers highlighted in blue indicate the antibiotic MIC and the phage cocktail MIM alone. Numbers highlighted in red indicate a shift in MOI and MIC for the phage/antibiotic combination. (**E**) FICI of the phage/antibiotic combinations. An FICI < 0.50 indicates a phage/antibiotic synergy, 0.50 ≤ FIC < 1.0 indicates an additive effect, 1.0 ≤ FICI < 2.0 indicates indifference, and FICI ≥ 2.00 indicates antagonism [31].

The MICs of the six antibiotics tested for Ab125 confirmed its XDR phenotype. In fact, only MIN showed an MIC ≤ 4 µg/mL, i.e., in the susceptibility range defined by EUCAST (Table S3). All other antibiotics harbored MICs far over the EUCAST breakpoints, with extreme values > 4000 and >5000 µg/mL for GEN and CIP, respectively (Table S3). These two very high MIC values were considered out of scope for our next experiments, so GEN and CIP were excluded from the rest of the study. Accordingly, we performed synograms on Ab125 with IPM, MIN, CTZ, and COL combined with the 3014/3098 phage cocktail. While the phage cocktail was found to have indifferent interactions with IPM (FICI = 2), MIN (FICI = 1.83), and CTZ (FICI = 2) (Figure 4A–C,E), an additive effect was detected with COL (FICI = 0.57) (Figure 4D,E).

**Figure 5:**
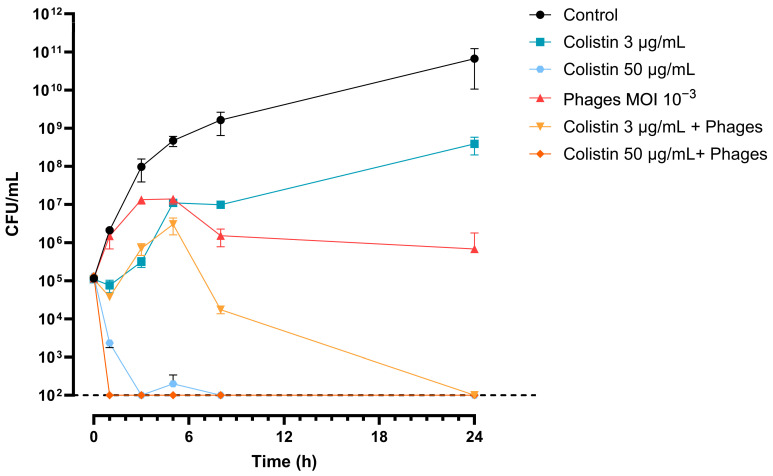
Time–kill assay with colistin and phage cocktail on Ab125. Detection limit is at 1 × 10^2^ CFU/mL (dashed line).

To investigate this effect further, we performed a time–kill assay with MOI = 1.00 × 10^−3^ for the phage cocktail and two concentrations of COL. While the phage alone showed bacteriostatic efficacy over 24 h (1.10 × 10^5^ ± 1.44 × 10^4^ CFU/mL at 0 h versus 1.33 × 10^5^ ± 5.77 × 10^4^ CFU/mL at 24 h, *p* = 0.3742), colistin at 3 μg/mL was not able to prevent bacterial growth when used alone (1.10 × 10^5^ ± 1.44 × 10^4^ CFU/mL at 0 h versus 3.91 × 10^8^ ± 1.92 × 10^8^ CFU/mL at 24 h, *p* = 0.0715). The combination was synergistic at 24 h with a >6 log10 CFU/mL decrease compared to the best treatment alone (no CFU detected versus 1.33 × 10^5^ ± 5.77 × 10^4^ CFU/mL at 24 h, for the combination and the phages alone, respectively, *p* < 0.05). When the concentration of COL increased to 50 μg/mL, synergy became apparent as early as 1 h post-challenge. Indeed, we observed a >3 log10 CFU/mL decrease in the bacterial load for the combination compared to COL alone, which was the most effective treatment performed alone in this case (no CFU detected versus 2.33 × 10^3^ ± 5.77 × 10^2^ CFU/mL at 1 h for the combination and COL alone, respectively, *p* < 0.05) (Figure 5).

## 4. Discussion

Treatment of *A. baumannii* infections is a major challenge, mainly due to the emergence of MDR and XDR strains [35]. Thus, investigations into innovative treatment strategies are needed to manage patients without therapeutic solutions [36]. In this study, we evaluated the efficacy of phage therapy against Ab125, an XDRAB strain collected from burn wounds. Phage vB_AbaM_3098 was the only phage found in our collection of 34 different lytic phages that could lyse Ab125. It is interesting to note that phage 3098 could belong to a new family of *A. baumannii* phages, as its genome harbored only very weak genome homology with phage vB_AbaM-SBP. Ab125 rapidly developed resistance in vitro under the selective pressure of phage 3098. Mechanisms behind the acquired phage resistance in *A. baumannii* are still poorly understood. For instance, it has been shown that a replicated 8 bp insertion, which increased in frequency within phage-resistant *A. baumannii* mutants, could result in early truncation of a protein of unknown function acting as a phage receptor [37]. Others found that capsule modulation provides *A. baumannii* with strategies to control phage infectivity for which the global regulator BfmRS is key. Mutations hyperactivating BfmRS simultaneously increase capsule levels, phage adsorption, replication, and host killing, while BfmRS-inactivating mutations reduce capsule synthesis and block phage infection [38]. Here, comparative genomics between Ab125 and Ab139 (i.e., an Ab125 clone resistant to phage 3098) highlighted three impactful variants: a deletion located in an intergenic region between two CDSs coding for hypothetical proteins, a T552G SNP (Asp184Glu) in a putative repressor protein belonging to the LexA family transcriptional regulator, and a Ser1267Asn variant in a cell surface trimeric autotransporter adhesin Ata. The LexA repressor is involved in the SOS response of bacteria, a coordinated cellular reaction to DNA damage, which is primarily regulated by the RecA and LexA proteins. In *Escherichia coli*, for instance, RecA facilitates the self-cleavage of the LexA repressor, leading to the induction of over 40 genes that constitute the SOS global regulatory network [39]. In certain well-characterized pathogens such as *A. baumannii*, activation of the SOS response influences the development and spread of drug resistance, as well as the synthesis, secretion, and dissemination of virulence factors [40]. Interestingly, LexA-like proteins have been shown to interact with phage gp7 proteins, thereby altering the dynamics of the cellular SOS response [41]. The Ata protein, a surface protein of *A. baumannii*, has been previously shown to bind components of the host cell’s extracellular matrix [42] and classified as a virulence factor [43]. We hypothesize that the Ata protein could represent a novel phage receptor in *A. baumannii*. Mutation of this protein could lead to alterations in the way the receptor-binding proteins (RBPs) of bacteriophages interact with the bacterium, resulting in resistance to phage vB_Aba_3098 and increased susceptibility to the six other phages in Ab139. A comparison of proteomic profiles identified one overexpressed and six underexpressed proteins in Ab139 compared to Ab125. However, we obtained no clear evidence for a potential link between the identified genomic variants and the proteomic pattern. Accordingly, deciphering the exact mechanism by which Ab139 acquired resistance to phage 3098 requires additional investigations. For instance, it remains to be elucidated whether the underexpression of the identified lytic murein transglycosylase B, known to be involved in cell wall biosynthesis, could be part of a phage-resistance mechanism. Indeed, it has been reported that mutation of a glycosyltransferase—which is related to transglycosylase—known to alter capsule structure and bacterial virulence, can cause complete phage resistance [38]. Similarly, it remains to be elucidated whether the underexpressed protein annotated as an RND-like efflux pump is linked to the phage resistance phenotype in *A. baummanni*, as is the case for other efflux pumps in *P. aeruginosa* [44]. However, and in contrast to other studies reporting increased sensitivity of phage-resistant clones to several antibiotics [44,45,46], we found no change in the antibiogram of Ab139 compared with Ab125. While it has previously been shown that phage resistance could significantly affect virulence, with phage-resistant variants successfully reducing mortality in a *G. mellonella* larval in vivo model [37,47], we did not observe a significant reduction or increase in in vivo virulence for Ab139. As a result, the resistance costs of these phages are most likely to be bacterial strain- and/or phage-dependent, justifying their systematic investigation. Strain Ab139 is significant due to its acquired susceptibility to six phages from our collection, including the *Lazarusvirus* phage 3014. For the mechanism of Ab139 resistance to phage 3098, further research is needed to decipher the exact mechanism by which Ab139 became susceptible to the six phages, while remaining otherwise inactive on Ab125. Both phages 3014 and 3098 fulfilled important criteria for therapeutic purposes, i.e., they are strictly lytic and do not carry genes encoding for antibiotic resistance determinants or virulence factors in their genomes [48]. Interestingly, the phage cocktail 3014/3098 was found to completely inhibit Ab125 growth in the turbidity assay. These in vitro results were confirmed in a *G. mellonella* model of infectious disease, supporting the in vivo potential of phage therapy against XDRAB infections. Currently, phages are mainly used as adjuvants in therapy protocols to standard of care (SoC) antibiotics, hence the importance of studying both in vitro and in vivo interactions before administering them to patients. While phage–antibiotic synergy (PAS) has often been reported as a means of radically improving bacterial inhibition and preventing the emergence of resistant strains [49,50,51,52,53,54], antagonisms, while less frequently reported, remain important as they can be detrimental to the host [54,55,56]. Accordingly, we tested the in vitro interaction of our phage cocktail with antibiotics, choosing those that represented SoC treatments in various *A. baumannii* infections at CHUV [57]. While no difference was found for most of the antibiotics tested, an additive interaction initially found in synograms for the phages/colistin combination became synergistic in time–kill assays as previously reported and explained by a structural change in the cell envelope [47]. In our study, the discrepancy in results between the synograms and time–kill assays might be linked to the different environments in which they were performed, i.e., soft-agar and liquid medium tests, respectively. Taken together, the results presented herein highlight the great potential of phage therapy to resist against XDRAB, as reported previously in preclinical [37,47,58,59,60,61] and clinical studies [15,62,63,64,65,66,67]. In addition, our work highlights how phages that were not initially active on the pathogenic strain proved highly valuable in a two-phage cocktail; they became highly active for resistant clones selected by the first phage. This observation aligns with the concept of step-by-step phage therapy outlined earlier, which aims to develop phage cocktails by initially and more systematically considering resistances that develop as phages are added to the cocktail [68]. Finally, our results demonstrate how the complexity of assembling cocktails of effective phages encourages systematic phagograms with as many phages as possible, not only on the pathogenic strain but also on emerging phage-resistant clones, particularly when these retain or increase their virulence in vivo.

## Figures and Tables

**Table 1:** Virulence index (Vp) and MV_50_ value for phages vB_AbaM_3014 (3014) and vB_AbaM_3098 (3098) on Ab125.

	Ab125		
	*V_P_*		*MV*_50_
Phage 3014	0		-
Phage 3098	0.47		-
Cocktail 3014/3098	0.62		1.00 × 10^−3^

## Data Availability

Bacterial WGS and sequencing reads for Ab125 and Ab139 are in NCBI BioProject PRJNA700858 under accession numbers (WGS/SRA): JBPURS000000000.1/SRX26916449, JBJOXQ000000000.1/SRX26916532, respectively. WGS and sequencing reads for phages 3014 and 3098 are in NCBI BioProject PRJNA691459 under accession numbers (WGS/SRA): PV081574.1/SAMN50004113, PV081573.1/SAMN50004114, respectively. The mass spectrometry proteomics data have been deposited to the ProteomeXchange Consortium via the PRIDE partner repository (https://www.ebi.ac.uk/pride/, accessed on 1 April 2025) with the dataset identifier PXD062593.

## Supplementary Materials

The following supporting information can be downloaded at: https://www.mdpi.com/article/10.3390/v17111441/s1. Figure S1: Virulence of XDRAB strains Ab125 and Ab139 in *Galleria Mellonella*, Figure S2: Virulence curves on Ab125, Table S1: List of non-synonymous variations, insertions and deletions found on the genomes of Ab139 clones when compared to Ab125, Table S2: Differentially expressed proteins in Ab139 versus Ab125, Table S3: MIC of various antibiotics for Ab125 and Ab139.
