# Supplementary material for: Rational Design of a Potent Two-Phage Cocktail Against a Contemporary Acinetobacter baumannii Strain Recovered from a Burned Patient at the Lausanne University Hospital
Source: Viruses. 2025 Oct 29;17(11):1441. doi: 10.3390/v17111441 (PMC12656882; doi:10.3390/v17111441)
Supplement: Supplementary file 1 [file viruses-17-01441-s001.zip › Figure S1.pdf]

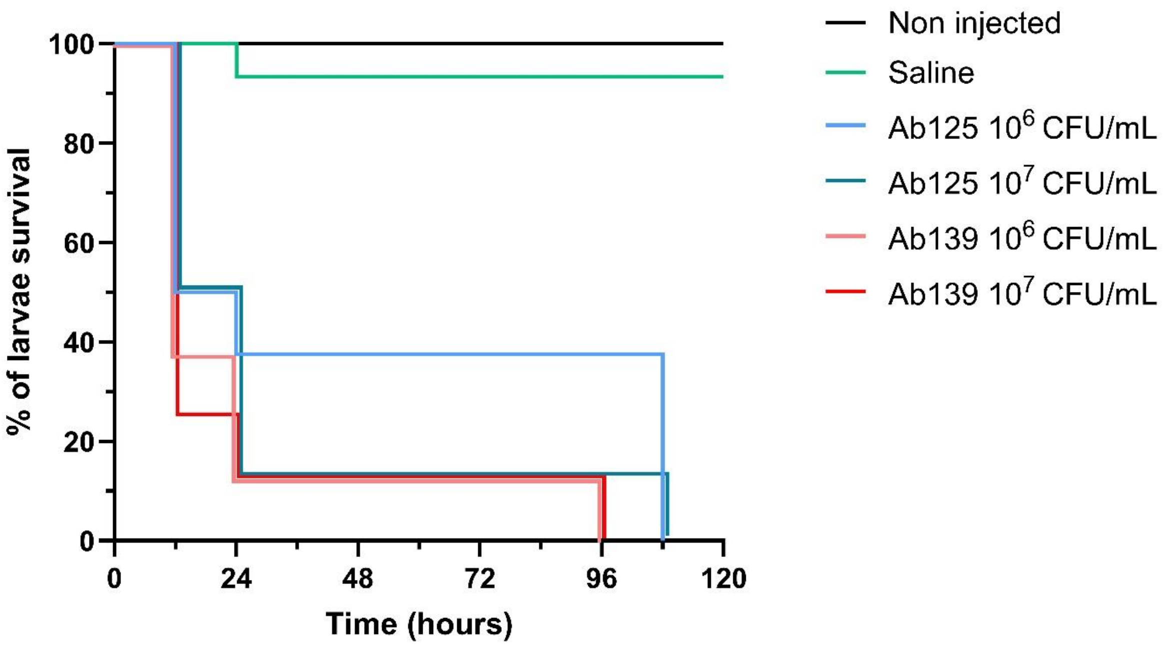

Figure S1. Virulence of XDRAB strains Ab125 and Ab139 in *Galleria Mellonella*. Percentage of survival is defined as the number of living larvae at a given time after injection divided by the total number of larvae prior to injection (N = 15). Control larvae were either not injected (non injected) or injected with NaCl 0.9% (Saline). Curves were compared with the log-rank (Mantel-Cox) and Gehan-Breslow-Wilcoxon tests.
