# Supplementary material for: Rational Design of a Potent Two-Phage Cocktail Against a Contemporary Acinetobacter baumannii Strain Recovered from a Burned Patient at the Lausanne University Hospital
Source: Viruses. 2025 Oct 29;17(11):1441. doi: 10.3390/v17111441 (PMC12656882; doi:10.3390/v17111441)
Supplement: Supplementary file 1 [file viruses-17-01441-s001.zip › Figure S2.pdf]

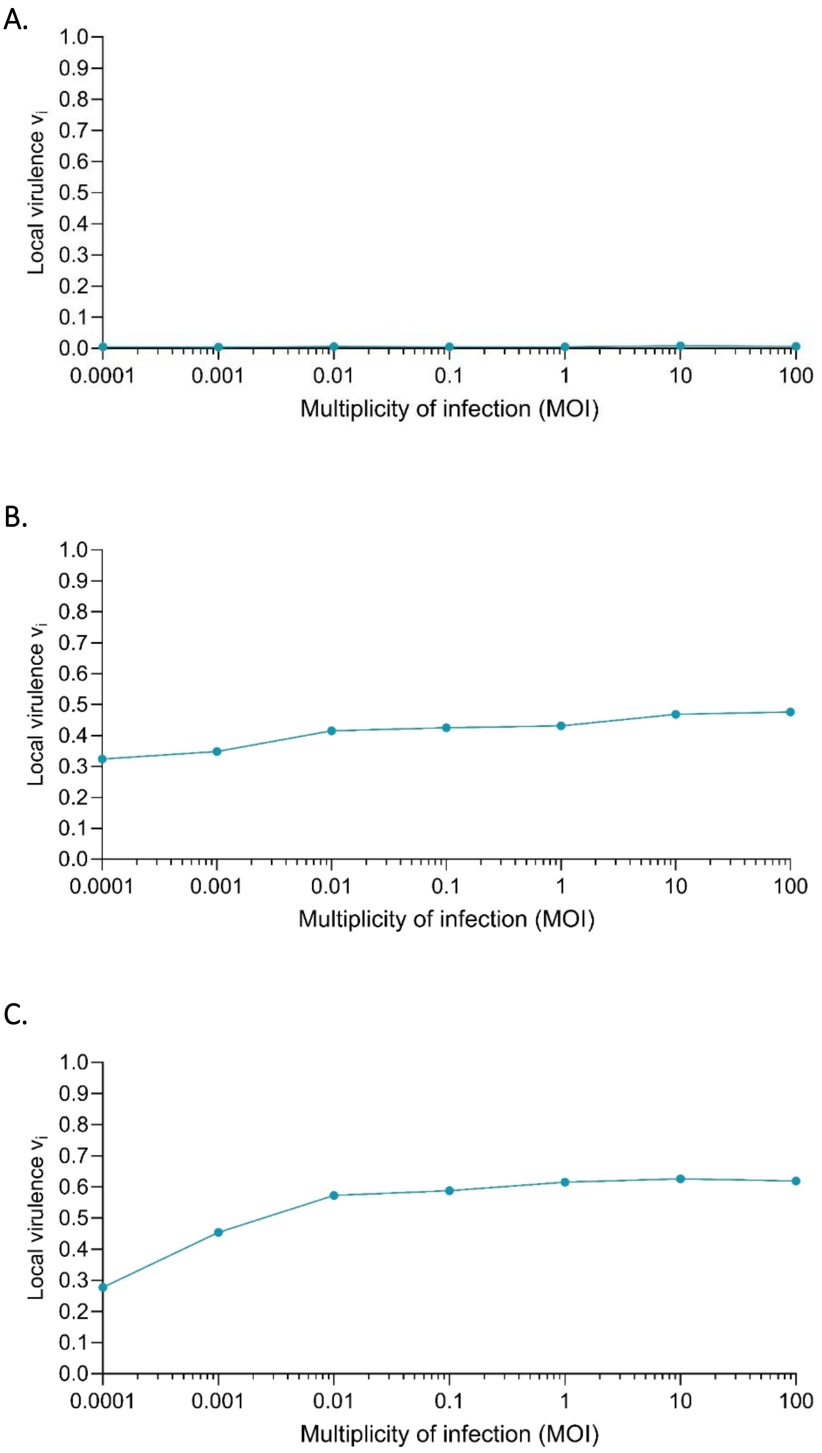

Figure S2. Virulence curves on Ab125. Virulence curves representing the local virulence (vi) in function of the multiplicity of infection (MOI) of phage **A.** vB_AbaM_3014, **B.** vB_AbaM_3098, and **C.** the combination of both on Ab125.
