# Supplementary material for: Rational Design of a Potent Two-Phage Cocktail Against a Contemporary Acinetobacter baumannii Strain Recovered from a Burned Patient at the Lausanne University Hospital
Source: Viruses. 2025 Oct 29;17(11):1441. doi: 10.3390/v17111441 (PMC12656882; doi:10.3390/v17111441)
Supplement: Supplementary file 1 [file viruses-17-01441-s001.zip › Table S1.pdf]

Table S1: Full list of non-synonymous variations, insertions and deletions found on the genomes of Ab139 clones when compared to Ab125. Variants shared by the six Ab139 clones are highlighted in red.

Pos	Ref	Var	Score	Var_cov	Type	Ref_nt_pos_change	Ref_aa_pos_change	Function	snpEff_type	snpEff_impact
142	C	T	760.101	690					intergenic_region	MODIFIER
163	G	A	313.611	728.3					intergenic_region	MODIFIER
5256	A	G	4444.39	917					intergenic_region	MODIFIER
5361	CAAG	TAAA	1336.31	803					intergenic_region	MODIFIER
5372	C	T	1959.05	797					intergenic_region	MODIFIER
22370	A	C	6599.38	261.7	Nonsyn	552T>G	Asp184Glu	putative repressor protein (LexA family transcriptional regulator)	missense_variant	MODERATE
36720	TACTAGCAAT	GACCAATAAC	3154.48	116	Nonsyn	3795_3804delTACTAGCAAT insGACCAATAAC	Ser1267Asn	Cell surface protein (trimeric autotransporter adhesin Ata)	missense_variant	MODERATE
42736	A	C	10828	339					intergenic_region	MODIFIER
12186	TAAAAAAAAGAGGAT	TAAAAAAAGAGGAT	3375.99	145	Deletion				intergenic_region	MODIFIER
