# Supplementary material for: Rational Design of a Potent Two-Phage Cocktail Against a Contemporary Acinetobacter baumannii Strain Recovered from a Burned Patient at the Lausanne University Hospital
Source: Viruses. 2025 Oct 29;17(11):1441. doi: 10.3390/v17111441 (PMC12656882; doi:10.3390/v17111441)
Supplement: Supplementary file 1 [file viruses-17-01441-s001.zip › Table S2.pdf]

Table S2: Differentially expressed proteins in Ab139 versus Ab125.

Mean Ab125.iBAQ.log2	Mean Ab139.iBAQ.log2	Protein names	Protein IDs	Gene names	p-value	Ab139 vs Ab125
-0.220898394	0.51334062	Acetyl-coenzyme A synthetase	A0A0E1JGH6	*acs*	4.73E-05	Overexpressed
-0.328928526	-1.096023929	Uncharacterized protein	A0A1E3M2H3	*AUO97_13765*	2.78E-05	Underexpressed
-1.009644483	-3.097627185	3-phosphate/5-hydroxy nucleic acid ligase	A0A1E3MBM1	*AUO97_03185*	1.41E-04	Underexpressed
2.523357144	-0.923821831	Tyrosine protein kinase	V5RDC9	*wzc*	9.71E-06	Underexpressed
4.279207041	3.214085761	DUF6091 family protein/RND type efflux pump	V5V8J3	*A7M90_19360*	1.24E-04	Underexpressed
0.303687637	-2.050159385	Polyadenylate binding protein human types 1;2;3;4 family	V5VGR3	*A7M90_00775*	1.70E-06	Underexpressed
1.777487017	0.549992317	Lytic murein transglycosylase B	A0A1E3M5Y7	*AUO97_00505*	2.11E-05	Underexpressed
