# Supplementary material for: Rational Design of a Potent Two-Phage Cocktail Against a Contemporary Acinetobacter baumannii Strain Recovered from a Burned Patient at the Lausanne University Hospital
Source: Viruses. 2025 Oct 29;17(11):1441. doi: 10.3390/v17111441 (PMC12656882; doi:10.3390/v17111441)
Supplement: Supplementary file 1 [file viruses-17-01441-s001.zip › Table S3.pdf]

Table S3. MIC of various antibiotics for Ab125 and Ab139.

**Antibiotic**	**Family**	**EUCAST MIC breakpoints (µg/mL)**		**Estimated MIC for Ab125 (µg/mL)**	**Estimated MIC for Ab139 (µg/mL).**
		**S ≤**	**R >**		
Minocycline	Tetracyclin	4	8	≤ 4; S	≤ 4; S
Colistin	Polymyxin	2	2	> 40; R	> 40; R
Imipenem	Carbapenem	2	4	> 50; R	> 50; R
Ceftazidime	Cephalosporin	16	16	> 200; R	> 200; R
Gentamicin	Aminoglycoside	4	4	> 4’000; R	> 4’000; R
Ciprofloxacin	Fluoroquinolone	0.001	1	> 5000; R	> 5000; R
